# Age Related Patterns of Disease and Mortality in Hospitalised Adults in Malawi

**DOI:** 10.1371/journal.pone.0168368

**Published:** 2017-01-18

**Authors:** Theresa J. Allain, Stephen Aston, Gugulethu Mapurisa, Thokozani N. Ganiza, Ndaziona P. Banda, Servace Sakala, Andrew Gonani, Robert S. Heyderman, Ingrid Peterson

**Affiliations:** 1 University Hospitals Bristol NHS Trust, Bristol, United Kingdom; 2 Malawi Liverpool Wellcome Trust Clinical Research Programme, Blantyre, Malawi; 3 College of Medicine, University of Malawi, Blantyre, Malawi; 4 Ministry of Health, Queen Elizabeth Hospital, Blantyre, Malawi; 5 University College London, London, United Kingdom; Liverpool School of Tropical Medicine, UNITED KINGDOM

## Abstract

**Background:**

The epidemic of non-communicable diseases (NCDs) in low and middle income countries (LMICs) is widely recognised as the next major challenge to global health. However, in many LMICs, infectious diseases are still prevalent resulting in a “double burden” of disease. With increased life expectancy and longevity with HIV, older adults may particularly be at risk of this double burden. Here we describe the relative contributions of infections and NCDs to hospital admissions and mortality, according to age, in Malawi’s largest hospital.

**Methods:**

Primary diagnosis on discharge/death, mortality rates, and HIV status were recorded prospectively on consecutive adult medical in-patients over 2 years using an electronic medical records system. Diagnoses were classified as infections or NCDs and analysed according to age and gender.

**Findings:**

10,191 records were analysed. Overall, infectious diseases, particularly those associated with HIV, were the leading cause of admission. However, in adults ≥55 years, NCDs were the commonest diagnoses. In adults <55 years 71% of deaths were due to infections whereas in adults ≥55 years 56% of deaths were due to NCDs.

**Interpretation:**

Infectious diseases are still the leading cause of adult admission to a central hospital in Malawi but in adults aged ≥55 years NCDs are the most frequent diagnoses. HIV was an underlying factor in the majority of adults with infections and was also present in 53% of those with NCDs. These findings highlight the need for further health sector shifts to address the double burden of infectious and NCDs, particularly in the ageing population.

## Background

Populations around the world are ageing, with the most dramatic increases in the number of older adults occurring in low and middle income countries (LMICs).[1] Although the population structure in sub-Saharan Africa (SSA) remains young in relative terms, this region already has double the number of older adults than northern Europe, and this figure is expected to grow faster than anywhere else in the world, increasing from 46 million in 2015 to 157 million by 2050.[2] Worldwide there is a growing burden of non-communicable diseases (NCDs) including cardiovascular diseases, chronic lung diseases, diabetes and cancer[3] along with a projected rapid shift from communicable diseases to NCDs as the dominant causes of morbidity and mortality in LMICs.[4] As NCDs predominate in middle and later life, older adults are likely to carry the greatest burden of NCDs. In addition, the successful roll out of antiretroviral therapy (ART) in many LMICs is leading to an increasing number of ageing adults living with HIV.[5] During this transition, it is feared that many LMICs will experience a double burden of disease with simultaneously high levels of both communicable diseases and NCDs.[6] The disease burden of older adults in LMICs has not been sufficiently studied.[7]

Though ranked among the youngest countries globally, Malawi’s population is rapidly ageing with an improvement in life expectancy from 37 to 58 years in the past decade[8] and an adjusted life expectancy of 77 years for those who live to age 60.[9] The number of adults aged over 60 years is projected to be >1 million by 2030 and >2 million by 2050.[1] Like many countries in SSA, Malawi is relatively early in the health transition, with rising rates of NCD risk factors such as obesity and hypertension[10] alongside a high burden of infectious diseases,[11] including an adult HIV prevalence of 10·6%.[12] In this context, it is likely that Malawi is experiencing a double burden of disease, which is preferentially affecting older adults, a group who are already experiencing complex health needs related to the co-morbidities of ageing.[7,13] We investigated this hypothesis by conducting an age-based analysis of medical inpatient surveillance data from the largest hospital in Malawi. The analysis may serve as a paradigm to guide healthcare policy aimed at better meeting the complex healthcare needs of the rapidly growing numbers of older people in LMICs.

## Methods

### Study setting and participants

Queen Elizabeth Central Hospital (QECH), Blantyre, Malawi is the largest government funded teaching and referral hospital in Malawi with over 7000 medical admissions per year. Medical care is provided free at the point of delivery. Diagnostic facilities are rudimentary but include smear microscopy and Gene Xpert MTB/RIF for tuberculosis. Blood and cerebrospinal fluid culture is provided by the Malawi Liverpool Wellcome Trust Clinical Research Programme (MLW). In accordance with Malawian national policy, provider initiated HIV testing and counselling (PITC) is offered to all hospitalised adults with unknown HIV status. However, owing to a lack of of HIV test kits or trained counsellors, there have been periods when PITC was not conducted.

### Hospital inpatient surveillance

The Surveillance Programme of IN-patients and Epidemiology (SPINE) database is an electronic medical record (EMR) that was established at QECH in 2009 as a collaboration between MLW and Baobab Trust under the auspices of the Malawi Ministry of Health (MOH).[11] In accordance with an MOH initiative to develop a fully digital Health Management Information System (HMIS) in government clinical facilities, this ward-based touch-screen system is used to record demographic and clinical information, vital status and HIV status on all patients admitted to the adult medical wards. At discharge or death, primary and secondary diagnoses are made on the basis of the available clinical, radiological and laboratory data. Diagnoses are selected from a list based on the HMIS diagnostic tree used in healthcare facilities throughout Malawi; the HMIS diagnostic tree was modified from the International Classification of Diseases (ICD-10). The SPINE data management team routinely undertakes systematic data quality checks to ensure the accuracy and completeness of data recorded in the SPINE system compared with the paper-based hospital HMIS which runs in parallel with SPINE. During the study period SPINE captured at least 100% of admissions/discharges, and 96% of deaths compared to HMIS.

### Procedures and statistical analysis

We analysed hospital inpatient episodes recorded in the SPINE system from 1^st^ January 2013 to 31^st^ December 2014 for all adults aged ≥16 years; among patients admitted multiple times during the study period only the first admission was included. In this study, the unit of analysis was hospital episode. Individual primary diagnoses were grouped according to the ‘root parent diagnostic group’ which are similar to the ICD-10 classification (S1 File). Diagnoses that were initially classified as ‘other’ were reviewed manually and when appropriate categorized under one of the other root parent diagnostic groups.

Whilst there is no universally accepted threshold for classifying a patient as elderly, the World Health Organization (WHO) suggests that in SSA an age threshold of 55 years may be used.[14] This further seems appropriate since the life expectancy in Malawi is currently 58 years.[8] Consequently the cohort of patients were divided into younger (<55 years) and older (≥55 years) patients. Hospital episodes were classified by primary diagnosis and tabulated in order of frequency. Primary diagnoses were then grouped into ‘Infections’, ‘NCDs’ and ‘Undefined’ (S1 File). For the 20 most common diagnoses, we examined the proportion of mortality by a binary variable for younger versus older age. A multivariable logistic model was developed to examine the effect of gender, disease type (‘NCD’,’Infection’,’Undefined’), HIV status and age on probability of death. In the model age was treated using cubic splines, which were selected over other spline formulas based on better model fit assessed through AIC value. Interactions by age and HIV, and age and disease type were examined in the model. Inpatient admission rates per 1000 population were calculated by dividing total recorded admissions (including readmission) by the population size within age/ sex strata; population data were obtained from the Malawi 2008 Census.[15]

### Ethical considerations

Since all clinical data used for this analysis were collected as part of the MOH HMIS during routine care, individual patient consent was not obtained. Our research team did not have contact with patients and only had access to fully anonymyzed data. These data were maintained by MLW’s Data Department using institutional data governance standard operating procedures which conform to international standards. This analysis was approved by the University of Malawi College of Medicine Research Ethics Committee.

## Results

**Fig 1:**
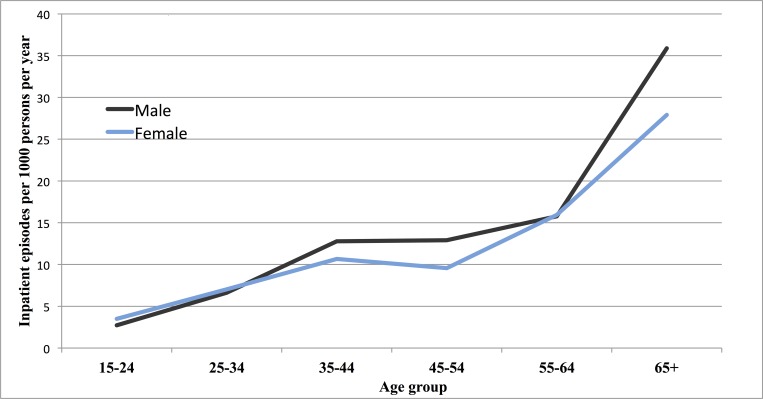
Annual incidence of inpatient admissions per 1000 population, by age and sex, Blantyre District, Malawi 2013–2015. The grey line represents ‘Male’, the light blue line represents ‘Female’.

Between 1^st^ January 2013 and 31^st^ December 2014, 11,645 patients were admitted to the QECH adult medical wards, of which 1133 (9·7%) were readmissions. In total, 184 and 137 patients were excluded from the analysis due to being <16 years old or having missing age data, respectively (S1 Fig). Our final data set included 10,191 first admission hospitalisations. The raw data file is available as supporting information (S2 File). Overall, 80·1% of admissions occurred in adults aged <55 years (n = 8161) compared to 19·9% in adults ≥55 years (n = 2030). However, the age specific rate of annual hospitals admission per 1000 population in Blantyre District rose steadily with increasing age (Fig 1).

### Differences in diagnosis by age

**Fig 2:**
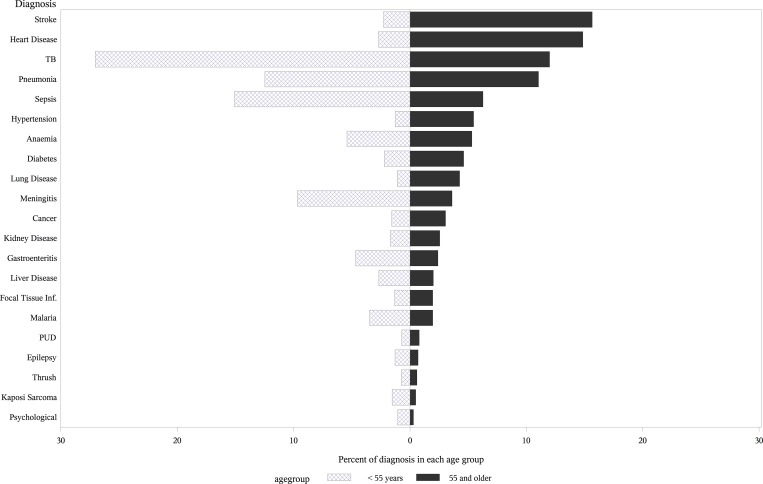
Distribution of diagnoses of adult patients by age category, Blantyre, Malawi, 2013–2014. The light grey hatched bars represent adults aged < 55 years; the solid dark grey bars represent adults aged 55 years and older.

**Fig 3:**
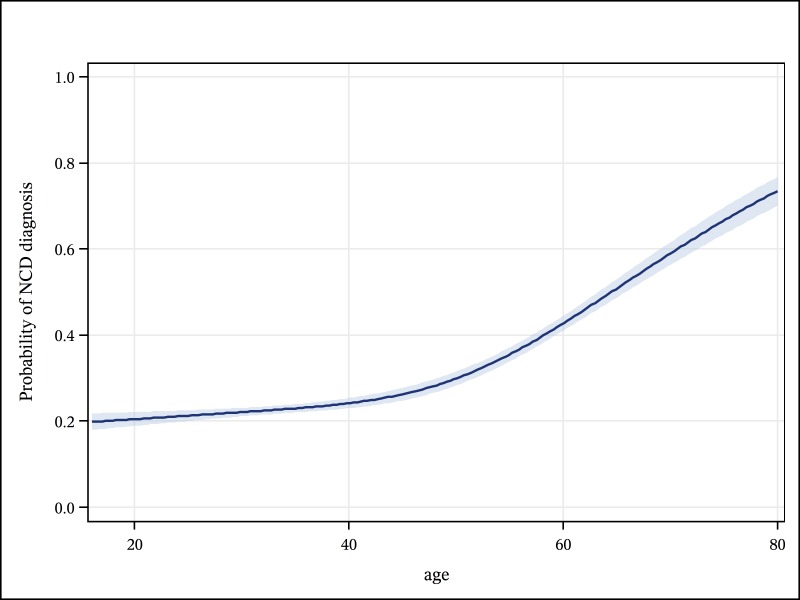
Predicted probablility (with 95% confidence limits) of a non-communicable disease diagnosis, by age, in adult inpatients, Blantyre, Malawi- 2013 to 2014.

Infectious diseases accounted for 6534/10191 (64·1%) of diagnoses, while NCDs accounted for 3052/10191 (29·9%) of diagnoses. Overall the most common diagnoses were tuberculosis (TB) (n = 2283, 22·4%), sepsis (n = 1265, 12.4%), pneumonia (n = 1162, 11·4%), and meningitis (n = 803, 7·9%) (Fig 2). In adults aged <55 years infections predominated, accounting for 70·9% of diagnoses. The top 4 diagnoses in this age group were TB, sepsis, pneumonia, and meningitis which together accounted for 60·3% of all diagnoses. Conversely, in older adults infections made up only 32·6% of diagnoses, while 55·7% of diagnoses were due to NCDs; stroke and heart disease were the two commonest diagnoses in this age group. The likelihood of an NCD diagnosis increased steadily with age (Fig 3). The major NCDs (heart disease, stroke, diabetes and hypertension) accounted for 37·9% of admissions in older adults and only 7·9% in younger adults.

### Mortality

**Fig 4:**
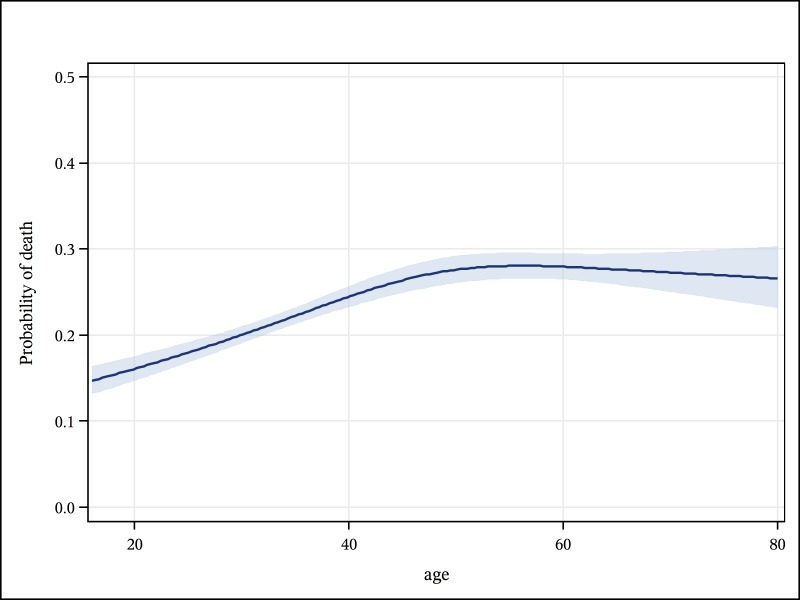
Predicted probablility (with 95% confidence limits) of a death, by age, in adult inpatients, Blantyre, Malawi- 2013 to 2014.

Overall mortality among hospital inpatients was 22·7%. The risk of death varied markedly by diagnosis; candidiasis, gastroenteritis, kidney disease, meningitis and cancer were associated with the highest mortality rates, ranging from 31·4% to 58·3% (Table 1). Patients aged ≥55 years had higher overall mortality than those aged <55 years (26·9 vs. 21·6%, p-value<·0001; Table 1); mortality rose to a peak of 27·6% in the 46–65 year age band, and thereafter plateaued (Fig 4). When analysed by diagnosis, death rates in older patients were higher for TB (36·8 vs. 29·4%), pneumonia (23·2 vs. 12·8%), sepsis (29.6 vs. 13·9%), and meningitis (44·4 vs. 33·0%).

**Table 1:** Mortality and case fatality rate by age and diagnosis (ranked by total mortality burden).

	All ages			under 55 years		over 55 years		Mortality difference by age P-value
**TB**	2283	689	30·2%	601	29·4%	88	36·8%	0·0181
**Meningitis**	803	273	34·0%	241	33·0%	32	44·4%	0·0498
**Sepsis**	1265	195	15·4%	158	13·9%	37	29·6%	<0·0001
**Pneumonia**	1162	172	14·8%	121	12·8%	51	23·2%	0·0001
**Heart**	500	132	26·4%	55	27·0%	77	26·0%	0·8133
**Stroke**	484	128	26·4%	45	26·2%	83	26·6%	0·9164
**Cancer**^**2**^	305	103	33·8%	76	32·5%	27	38·0%	0·3865
**Other Diagnosis**	534	101	18·9%	77	18·2%	24	21·4%	0·4446
**Anaemia**	515	87	16·9%	63	15·4%	24	22·6%	0·0763
**Liver Disease**	242	85	35·1%	70	34·7%	15	37·5%	0·7304
**Gastroenteritis**	401	74	18·5%	61	17·3%	13	27·1%	0·1005
**Kidney**	179	69	38·5%	44	34·4%	25	49·0%	0·0692
**Diabetes**	259	45	17·4%	28	16·8%	17	18·5%	0·7279
**Candidiasis**	68	32	47·1%	25	44·6%	7	58·3%	0·3886
**Malaria**	301	33	11·0%	27	10·3%	6	15·4%	0·4063^3^
**Hypertension**	205	29	14·1%	13	13·5%	16	14·7%	0·8157
**No Diagnosis**	78	24	30·8%	11	20·0%	13	56·5%	0·0014
**Focal tissue infection**	140	17	12·1%	10	9·9%	7	17·9%	0·1973
**Lung Disease**	168	14	8·3%	7	8·4%	7	8·2%	0·9629
**Peptic Ulcer Diease**	70	7	10·0%	4	7·4%	3	18·8%	0·3387^3^
**Epilepsy**	111	6	5·4%	6	6·2%	0·	0·0%	0·9999^3^
**Psychological**	86	2	2·3%	0	0·0%	2	33·3%	0·0041^3^
**Alcoholism**	32	0	0·0%	0	0·0%	0	0·0%	0·0834^3^
**Total**	10191	2317	22·7%	1743	21·6%	574	26·9%	<0·0001

^1^48Patients who absconded were classified as alive

^2^Including Kaposi Sarcoma

^3^ Fischer’s Exact Test

The proportion of mortality due to NCDs and infectious disease differed by age. In adults aged <55 years, 1270/1743 deaths (70·9%) were due to infection, while only 403 (23·1%) of deaths were due to the NCDs. In adults aged ≥55 years, infections accounted for 246/574 (38·2%) of death and NCDs accounted for 286/574 (55·7%) of deaths.

HIV status, gender and disease type significantly influenced risk of death (Table 2). Mortality in HIV-infected patients was 25·0% compared to 13·7% in HIV-uninfected patients. In multivariable analysis controlling for age, gender and disease type, HIV infected patients had double the risk of death, compared to HIV uninfected patients. Infectious disease diagnoses were associated with slightly higher risk of death compared to NCDs and male inpatients had 40% higher risk of death compared to female inpatients (Table 2).

**Table 2:** Demographic and clinical characteristics and risk of death among adult inpatients at QECH 2013–2014.

			Unadjusted		Adjusted	
	Total number	Deaths^1^ N(%)	OR	(95% CI)	OR	(95% CI)
**Total**	10191	22·7				
**HIV status**						
**Non-Reactive**	1489	204 (13·7)	1·0	—	1·0	—
**Reactive**	4551	1132 (25·0)	2·1	(1·8, 2·5)	2·1	(1·7, 2·5)
**Unknown**	4151	976 (23·5)	1·9	(1·6, 2·3)	1·8	(1·5, 2·1)
**Gender**						
**Female**	5071	994 (19·6)	1·0	—	1·0	—
**Male**	5120	1378 (26·9)	1·4	(1·3, 1·6)	1·4	(1·3, 1·5)
**Age group**						
**16–25**	1741	276 (15·8)	1·0	—	1·0	—
**26–35**	3007	626 (20·8)	1·4	(1·2,1·6)	1·2	(1·1, 1·4)
**36–45**	2237	546 (24·4)	1·7	(1·5, 2·0)	1·4	(1·2, 1·7)
**46–55**	1176	325 (27·6)	2·0	(1·7, 2·4)	1·8	(1·5, 2·2)
**56–65**	903	250 (27·6)	2·0	(1·7, 2·5)	2·0	(1·7, 2·5)
**> 65**	1127	298 (26·4)	1·9	(1·6, 2·3)	2·0	(1·7, 2·5)
**Disease Class**						
**NCD**	3052	638 (20·9)	1·0	—	1·0	—
**Infectious**	6534	1516 (23·2)	1·1	1·0, 1·3	1·2	(1·1, 1·3)
**Undefined**^**2**^	605	161 (26·6)	1·3	1·1, 1·7	1·4	(1·2, 1·8)

^1^ 48 Patients who absconded were classified as alive

2 Undefined cases were predominately complex cases where both infectious diseases and NCDs were present

### HIV status

**Fig 5:**
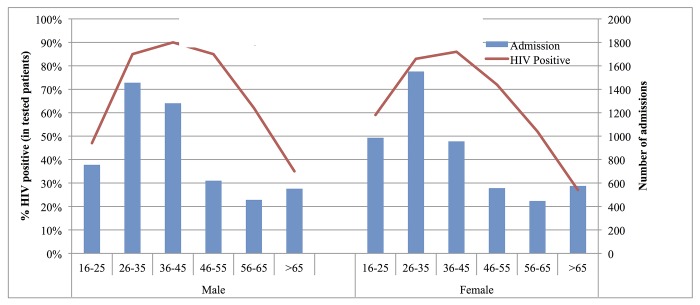
HIV Prevalence by age and sex in medical ward in-patients, Blantyre, Malawi, January 2013- December 2014. The red line represents the percent of admitted patients that are HIV infected. The blue bars represent the number of patients admitted.

In total, 4317 (42·3%) of patients had a recorded HIV status upon admission; a further 1431 (14·0%) had a new HIV test during their inpatient stay. HIV prevalence peaked at approximately 90% in the 36–45 year age group in both sexes (Fig 5). Among those tested HIV prevalence was significantly higher in adults aged <55 years compared to those aged ≥55 years (82·6% vs. 53·3%; p-value <0·001).

Age, gender and diagnosis influenced likelihood of having a known HIV status upon admission and having a new HIV test during the inpatient stay. In multivariable analysis controlling for age, sex and diagnosis, male patients (aOR = 0·59, 95% CI: 0·55, 0·65) and patients aged ≥55 years (aOR = 0·34, 95% CI: 0·29, 0·38) were less likely to be admitted with a documented HIV status. They were also less likely to be tested during the admission; the likelihood of having a new HIV test during inpatient stay was lower in adults aged ≥55 years (aOR = 0·56, 95% CI: 0·47, 0·66) and in men (aOR = 0·72, 95% CI: 0·63,0·82)(data not shown). On discharge 31·8% of older adults knew their HIV status compared to 62·9% of younger adults, p-value<0·0001 (Table 3).

**Table 3:** HIV testing and status by age and diagnosis in adult inpatients at QECH, 2013–2014, ranked by total proportion HIV positive.

		HIV status known						HIV % positive of those tested	
Diagnosis	Total admissions	All Ages		under 55		over 55		under 55	over 55
	N	N	%	N	%	N	%		
**Kaposi’s sarcoma**	126	103	81·7%	100	86·2%	3	30·0%	100·0%	100·0%
**Candidiasis**	68	49	72·1%	44	78·6%	5	41·7%	100·0%	100·0%
**Gastroenteritis**	401	246	61·3%	230	65·2%	16	33·3%	91·3%	75·0%
**Tuberculosis**	2283	1781	78·0%	1641	80·3%	140	58·6%	91·3%	65·7%
**Meningitis**	803	509	63 4%	476	65·1%	33	45·8%	88·9%	87·9%
**Pneumonia**	1162	656	56·5%	583	61·9%	73	33·2%	89·0%	65·8%
**Sepsis**	1265	668	52·8%	626	54·9%	42	33·6%	75·9%	70·9%
**Alcoholism**	32	4	12·5%	4	13·8%	0	0·0%	75·0%	—
**Liver Disease**	242	129	53·3%	117	57·9%	12	30·0%	75·2%	41·7%
**Other**	534	282	52·8%	244	57·8%	38	33·9%	71·7%	60·5%
**Malaria**	301	126	41·9%	119	45·4%	7	17·9%	68·9%	42·9%
**Anaemia**	515	300	58·3%	264	64·5%	36	34·0%	67·8%	58·3%
**Kidney**	179	92	51·4%	69	53·9%	23	45·1%	68·1%	60·9%
**Peptic Ulcer Disease**	70	38	54·3%	32	59·3%	6	37·5%	65·6%	66·7%
**No Diagnosis**	78	46	59·0%	37	67·3%	9	39·1%	75·7%	22·2%
**Diabetes**	259	72	27·8%	55	32·9%	17	18·5%	60·0%	52·9%
**Hypertension**	205	58	28·3%	38	39·6%	20	18·3%	65·8%	40·0%
**Cancer**	179	78	43·6%	62	52·5%	16	26·2%	66·1%	12·5%
**Focal Tissue Infection**	140	71	50·7%	58	57·4%	13	33·3%	56·9%	30·8%
**Lung disease**	168	57	33·9%	32	38·6%	25	29·4%	65·6%	32·0%
**Epilepsy**	111	28	25·2%	25	25·8%	3	21·4%	48·0%	33·3%
**Stroke**	484	163	33·7%	90	52·3%	73	23·4%	61·1%	26·0%
**Heart**	500	159	31·8%	94	46·1%	65	22·0%	56·4%	21·5%
**Psych**	86	33	38·4%	31	38·8%	2	33·3%	41·9%	0·0%
**Total**	10191	5748	56·4%	5071	62·9%	677	31·8%	82·6%	53·3%

Patients with an infectious disease diagnosis were more likely to have a documented HIV status on admission compared to patients with an NCD diagnosis, (aOR = 1·69, 95% CI: 1·54,1·86) and were 44% more likely than those with an NCD diagnosis to receive an HIV test during their hospital stay (aOR = 1·44, 95% CI: 1·26,1·67) (data not shown). Among those who were tested, 83·4% with an infectious diagnosis and 53·3% with an NCD diagnosis were HIV infected.

## Discussion

In an inpatient surveillance dataset of over 10,000 consecutive adult admissions from a large urban hospital in SSA, we found a double burden of infections and NCDs contributing to hospital admissions and in-patient mortality. Although infectious diseases were the dominant reason for admission, NCD diagnoses represented nearly one third of all admissions, and predominated in older adults, in whom over half were treated for an NCD. Comorbidity with HIV had a strong influence on our patient cohort, across all age groups and diagnoses. More than half of patients aged ≥55 years with a known HIV status were HIV positive and, across all ages, among those diagnosed with an NCD, the HIV prevalence was over 50%. The pattern of diagnoses for both infectious and non-infectious diseases are consistent with other hospital based surveys from the region [16,17] and with the global burden of disease literature which describe TB and respiratory infections as the main infections in adults, and stroke and heart disease as the main NCDs.[18] Our data adds to a limited but growing body of evidence showing that the rising trend of NCDs is impacting high HIV prevalent SSA countries, such as Malawi, where an ageing population are suffering the double burden of infections, including HIV, and NCD co-morbidity.

Our data highlight that more than a decade following the scale-up of ART, HIV remains an important driver of inpatient admission and mortality in this setting. While WHO recommends routine HIV testing in clinical settings, in our cohort only 43% of inpatients knew their HIV status on admission, and only 24% of patients with an unknown HIV status received an HIV test during their hospital stay. These rates are similar to those reported in other inpatient settings in SSA[19] and underline inadequate access to HIV testing in community and clinical settings.[20] Men, older adults and patients with an NCD diagnosis were less likely to have known HIV status on admission or be tested in-hospital. This variation in testing practice may be due to the misconception by both patients and clinicians that older adults and those with NCDs are at low risk of HIV. Higher rates of HIV testing in women compared to men has been noted previously.[21] Overall, 44% of patients in our cohort were discharged without knowing their HIV status. Although we were unable to assess the occurrence of post-discharge HIV testing, we note that there is currently no mechanism in place to facilitate such testing in this high risk group.

Although essential at all ages, strengthening the HIV spectrum of care from HIV testing to ART, and interventions within HIV clinics to address opportunistic infections[22] may be particularly important in older adults. HIV disease has been found to progress more rapidly in older people,[23,24,25,26] and, in developed countries people with HIV infection may have up to five times the risk of chronic diseases, geriatric syndromes and multimorbidity, even when the HIV infection is well treated and managed.[27] In our study, older adults had higher disease specific mortality rates than younger adults for the major infections associated with HIV.

Inpatient mortality was over 20%; predictors of death were HIV infection, male gender and older age. Alongside HIV, our data revealed that NCDs play an important primary or contributing role to inpatient mortality in older people in settings such as ours. The leading causes of mortality in over 55s (56% of deaths) were due to NCDs. These data underscore the need for all countries undergoing the health transition to develop NCD strategies including prevention as well as chronic care and acute clinical services orientated towards NCD care. The WHO have defined the minimum standards of care for non-communicable diseases in low resource settings.[28] There is also a need for greater integration of HIV/NCD chronic care, which at a minimum might include routine NCD and HIV screening (HIV screening in NCD clinics and NCD screening in HIV clinics) to reduce late presentation of illness to hospital, as well as combined care clinics.

The challenge of strengthening the HIV spectrum of care, scaling up routine HIV/NCD screening, and achieving greater integration of NCD/HIV clinical services need to be addressed at many levels. Medical education in SSA needs to be reoriented to prepare clinicians to respond to the changing patterns of disease; investment in public health for prevention and screening is needed, restructuring of chronic care and specialist expertise and appropriate specialist services need to be developed. The Malawi Ministry of Health recognises these challenges and is attempting to address them, for example through the development of dialysis units in the government hospitals in Lilongwe and Blantyre[29], pilot projects of integrated NCD/HIV in community sites [30] and national scale-up of NCD services, especially for diabetes and hypertension. However, in a severely resource constrained setting large-scale implementation of many of these measures may be difficult.

Our study has several limitations. Diagnostic resources were limited so many diagnoses were based on clinicians’ judgement using available evidence, although diagnoses of blood stream infection and meningitis where supported by good quality microbiology from the MLW programme. We only considered primary diagnoses but older patients may have multiple comorbidities which were not captured by this approach. The allocation of some diagnoses into infectious disease and NCD categories was also challenging. For example, many cancers in our setting are initiated by infections (e.g. cervical cancer and Kaposi’s sarcoma). These were classified as NCDs, which obscures the true complexity of the disease aetiology. With some diagnoses, for example chronic liver disease secondary to hepatitis infection, a decision was made not to classify the condition as infection or NCD which accounts for the “undefined” category (S1 File). Our analysis was restricted to first admissions and thus may have under estimated mortality which may be more common in readmitted patients. We were unable to prevalence of NCD risk factors, including smoking, as these are not captured in the SPINE system. Despite these limitations our results are likely to be unbiased as diagnostic data were collected independently in real time, and disease classifications followed pre-established rules developed prior to data analysis.

In conclusion, we have described primary diagnosis, mortality and prevalence of HIV infection in >10,000 Malawian adults admitted over two years at a Malawian central hospital using systematically collected, prospective data. Our age-based analysis demonstrates the complexity of the health needs of the older population. Infections, particularly HIV-associated infections, remain the dominant reason for admission, however, there is an equal balance of NCDs and infections in adults aged over 55, in whom HIV testing rates were considerably lower than in younger adults despite high HIV prevalence. With increased life expectancy, including in the HIV-infected population, LMICs which are poised for a shift in health policy focus toward NCDs must prepare, in the interim, for the challenges of the double burden of both infections and NCDs, particularly in older adults.

## Supporting Information

S1 File Definitions for diagnostic categories. (DOCX)

S2 File Diagnoses list. (XLS)

S1 Fig Study consort diagram for analysis of age related patterns of disease and mortality in hospitalised adults in Malawi. (DOCX)
