# Supplementary material for: Age Related Patterns of Disease and Mortality in Hospitalised Adults in Malawi
Source: PLoS One. 2017 Jan 18;12(1):e0168368. doi: 10.1371/journal.pone.0168368 (PMC5242517; doi:10.1371/journal.pone.0168368)
Supplement: S1 File — (DOCX) [file pone.0168368.s001.docx]

**Appendix 2 Definitions for diagnostic categories**

General principle based on hierarchy of presentation: Anaemia, ART failure, immunosuppression, hypoglycaemia, candidiasis, chronic diarrhoea, hypertension, arthritis are overridden by alternative primary or secondary diagnosis if likely to be more acute.

Oesophageal candida as a sole primary diagnosis cannot be overridden by a secondary

TB = Infectious disease

All active TB including pulmonary and extrapulmonary as primary diagnosis. Includes TB meningitis and TB pericardial effusion.

Includes definite and clinical diagnoses of TB

TB as a secondary diagnosis when the primary diagnosis is pneumonia, pleural effusion or adenopathy

TB as a secondary diagnosis when the primary diagnosis could be a cause or complication of TB eg anaemia, ART failure

Not post infective complications eg lung fibrosis

Pneumonia= infectious disease

All cases where pneumonia/ chest infection/URTI or LRTI/PCP was the sole primary diagnosis. Includes cases where pneumonia is primary diagnosis with positive BCs.

Pneumonia as a secondary diagnosis if the primary diagnosis was an underlying cause (eg immunosuppression) or a less acute diagnosis eg anaemia, hypertension or if primary diagnosis is sepsis.

Primary diagnosis was a complication of pneumonia eg empyema or lung abscess

Includes aspiration pneumonia and HAP where these are the sole primary diagnosis and not due to stroke

If NTS blood stream infection in association with pneumonia as secondary diagnosis coded as NTS/Pos BC

If malaria as primary diagnosis with pneumonia as secondary diagnosis code as malaria

If malaria and pneumonia = first diagnoses code as pneumonia

If pneumonia = first diagnosis with chronic complication of HIV eg chronic gastroenteritis or candidiasis code as pneumonia.

If pneumonia joint first diagnosis with chronic lung disease (asthma, COPD, bronchiectasis) code as lung disease.

Meningitis= Infectious disease

All primary diagnoses of meningitis. Causes include bacterial, cryptococcal, viral and unspecified.

TB meningitis classified with TB.

Cerebral malaria with secondary diagnosis of meningitis classified as malaria.

Primary diagnosis of uncomplicated malaria with joint primary or secondary diagnosis of meningitis classified as meningitis.

Encephalitis is classified as other

Sepsis as primary but meningitis as joint primary or secondary =meningitis

Meningitis as secondary diagnosis with subacute condition eg anaemia, candida or hypertension as primary diagnosis

Malaria= infectious disease

All acute malaria including complicated malaria

Malaria as primary diagnosis with sepsis as primary or secondary diagnosis

Malaria as primary diagnosis with gastroenteritis as primary or secondary diagnosis

Malaria as a secondary diagnosis when primary diagnosis can be a complication of malaria

HSM

Acute/chronic anaemia attributed to malaria classified as malaria

Anaemia= NCD

Anaemia with a primary haematological (non-malignant), diagnosis, drug induced or IDA with no other cause or unspecified anaemia with no other cause. Includes sickle cell and thalassaemia.

In multiple worksheet, episodes usually classified as alternative diagnosis except when in combination with secondary diagnoses not causally related to anaemia or that are apparent complications of anaemia

Kaposi=NCD, combine with cancer for main analysis but note number of KS cases

All KS as a sole primary or secondary diagnosis including direct complications of KS such as cellulitis

KS as joint primary or secondary diagnosis coded as kaposis if other primary diagnosis is subacute eg anaemia, hypertension, immunosupression

KS as joint primary diagnosis with other important diagnoses eg pneumonia, oesophageal candida or TB coded as multiple

Kidney= NCD

Acute or chronic renal failure without a cause specified

If joint primary or secondary diagnoses, classified as alternative diagnosis if renal failure likely causally related (e.g. sepsis, pneumonia, gastroenteritis).

Classified as kidney if additional diagnosis likely consequence of renal failure e.g. anaemia.

Nephrotic syndrome

Nephrolithiasis

Renal tract infections coded as UTI/organ infection

Renal failure as a diabetic complication coded as diabetes

Stroke=NCD

Any case where primary diagnosis was stroke, CVA or hemiplegia but not a SOL or cerebral infection

Including ischaemic, haemorrhagic and SAH

Excludes primary diagnosis of stroke where primary or secondary diagnosis was meningitis- coded as meningitis

Any case where stroke was a joint primary or secondary diagnosis but the primary diagnosis was likely to be a risk factor eg HT or DM or a complication, eg aspiration pneumonia

Heart disease=NCD

Includes all references to heart or cardiac failure/insufficiency or cardiomyopathy as primary diagnosis. Also includes: acute coronary syndromes (angina, myocardial infarction, ischaemic heart disease); arrhythmias (atrial fibrillation, heart block); valvular heart disease (rheumatic or not specified)

Heart failure as secondary diagnosis if primary diagnosis likely to be a cause eg HT

Cor pulmonale and right ventricular failure as primary diagnosis

Infective endocarditis classified under organ infection

Cor pulmonale included here also. Hypertension alone classified as hypertension, but if heart disease mentioned included here.

Pleural effusion included here if heart failure mentioned, otherwise to other.

Pericardial effusion included here unless specified as TB or malignant.

Hypertension=NCD

Any case where the primary diagnosis was HT or the secondary diagnosis was HT but the primary diagnosis was likely to be a consequence of HT eg encephalopathy (but excluding stroke).

In the case of multiple diagnoses, the joint primary or secondary diagnosis was considered more likely to be the acute cause of admission.

Diabetes=NCD

All references to diabetes, ketoacidosis, HONK as sole primary diagnosis.

If diabetes a joint diagnosis with acute alternative presentation coded as alternative diagnosis eg TB, pneumonia

Includes hypoglycaemia unless alternative primary diagnosis specified.

Includes complications such as ulcers.

Hypertension as a primary diagnosis with diabetes as a secondary diagnosis coded as hypertension.

Alcohol =NCD

Intoxication and immediate complications as a sole primary diagnosis go to alcohol

Cirrhosis coded as liver, cardiac complications as hrt

Gastroenteritis= infectious disease

Any case where the sole primary diagnosis was acute or chronic gastroenteritis (unless NTS was the causative organism identified in BC).

All chronic diarrhoea as sole primary diagnosis. If joint primary or secondary coded as gastro only if alternative diagnosis considered to be less acute.

Dysentry and infective colitis

No mention of sepsis as a primary or secondary diagnosis

Cases where the secondary diagnosis was GE where the primary diagnosis was likely to be a consequence of GE eg hypoglycaemia, acute abdomen

Peptic ulcer disease=NCD

Any gastritis, PUD, GI bleeding as primary diagnosis

Above as secondary diagnosis if primary diagnosis non-specific, eg “ART failure”

Excludes varices (coded as liver)

Psych=NCD

All cases where confusion was the primary diagnosis with no obvious underlying cause eg sepsis.

Psychosis, depression, dementia, hysteria, psychosomatic as primary diagnosis

Epilepsy=NCD

All cases where epilepsy was primary diagnosis. Grand mal petit mal focal and unspecified.

All cases where seizures (focal or generalised) were the primary diagnosis with no attributable secondary cause

UTI=infectious disease, renal tract infections : recode in organ infection

UTI or pyelonephritis as primary diagnosis

UTI as secondary diagnosis where primary diagnosis was a subacute condition eg OA

Serious infections/Sepsis 3 categories, all infections.

1. NonTyphoid Salmonella/Positive Blood Cultures

NTS isolated in any severe infection

Diagnosis of “malaria” but positive BC

Sepsis/typhoid, organism isolated

Non focal infections with positive BCs

Positive blood cultures for *S Pneumoniae* isolated in pneumonia and meningitis coded as pneumonia/meningitis

1. Sepsis

Diagnosis of typhoid or clinical sepsis with no focus. No culture results.

1. Other infections: Acute organ infection- serious localised infections, usually bacterial but not meningitis/pneumonia eg renal tract infections, liver abscess, biliary, osteomyelitis, pelvic inflammatory disease, cellulitis, tonsillitis, endocarditis

Lung disease= NCD

COPD, asthma, bronchiectasis, fibrosis as primary diagnosis regardless of a joint primary or secondary diagnosis of pneumonia/bronchopneumonia

Pneumothorax

Pulmonary hypertension coded as other

Corpulmonale/RVF as sole primary diagnosis coded as heart

Corpulmonale/RVF as joint primary diagnosis with chronic lung disease coded as lungdz

Pleural effusion with no qualifying diagnosis coded as other

Thrush=candida =infectious disease

All cases where candida, thrush or oesophageal candida are the sole primary diagnosis

Cases where oesophageal candida is a joint primary diagnosis will be coded as the other primary diagnosis if they are meningitis, pneumonia, or culture positive sepsis

Paraplegia= NCD Put in “other” as such a small group

All non malignant/non-TB paraplegia

Liver=Not classified as infection or NCD

Acute and chronic liver disease, including decompensated liver disease unless due to malignancy or focal infection

Includes acute and chronic hepatitis (viral, drug induced, idiopathic), cirrhosis, jaundice (hepatic or posthepatic)

Ascites if no other cause specified eg TB malignancy heart failure or renal failure

Jaundice if no other cause specified

Varices and portal hypertension
