# Supplementary material for: Age Related Patterns of Disease and Mortality in Hospitalised Adults in Malawi
Source: PLoS One. 2017 Jan 18;12(1):e0168368. doi: 10.1371/journal.pone.0168368 (PMC5242517; doi:10.1371/journal.pone.0168368)
Supplement: S1 Fig — (DOCX) [file pone.0168368.s003.docx]

# Study consort diagram for analysis of age related patterns of disease and mortality in hospitalised adults in Malawi

*Excluded*

*Excluded*

*Excluded*

10191 included in the final analysis

10328 patients aged > 15 years

10,512 patients

(first admissions)

11,645 admissions to adult medical wards, 2013-2014
